# Therapeutic approaches for endocrine dysregulation in anorexia nervosa in adolescents

**DOI:** 10.3389/fped.2026.1765684

**Published:** 2026-03-19

**Authors:** Lotte Hoskens, Lieve Sevenants, Margaux Van Insberghe, Kristina Casteels

**Affiliations:** 1 Department of Pediatrics, University Hospitals Leuven, Leuven, Belgium; 2 Department of Development and Regeneration, KU Leuven, Leuven, Belgium

**Keywords:** adolescents, anorexia nervosa, bone mineral density, endocrine dysfunction, growth, pubertal delay

## Abstract

Anorexia nervosa (AN) is a complex eating disorder characterized by self-induced weight loss, distorted body image, and profound endocrine dysregulation. In adolescents, the illness leads to growth failure, pubertal delay, amenorrhea, and compromised bone mineral accrual. This mini review synthesizes current evidence regarding the pathophysiology and management of endocrine disturbances in adolescents with AN, with a particular focus on therapeutic strategies for growth failure, pubertal arrest, and reduced bone mineral density. Endocrine abnormalities in AN involve adaptive suppression of the hypothalamic–pituitary–gonadal, –adrenal, –thyroid, and –growth hormone axes. Nutritional rehabilitation remains the cornerstone of recovery, as restoration of energy balance normalizes gonadotropin secretion, promotes catch-up growth, and improves bone accrual. Among hormonal options, physiologic transdermal estrogen is currently the only evidence-based strategy for improving bone density and supporting reproductive recovery. Adjunctive therapies such as rhGH, rhIGF-1 or DHEA may hold potential in specific subgroups but require further validation through larger, age-specific randomized trials.

## Introduction

Anorexia nervosa (AN) is a severe eating disorder characterized by low body weight, distorted body image, and restrictive caloric intake. Epidemiological data indicate a lifetime prevalence ranging from 0.3% to 1.5% in females and 0.1% to 0.5% in males (1). Beyond its profound psychological and nutritional consequences, AN is associated with significant complications in adolescents, including delayed puberty, amenorrhea, stunted growth, and reduced bone mineral density (BMD).

The pathophysiology of this hormonal dysregulation in AN is multifactorial with nutritional deficiency serving as the primary contributing factor.

This mini review provides a practical overview of endocrine consequences and therapeutic considerations in adolescents with anorexia nervosa. Although the disorder also affects males, most pediatric endocrine evidence concerns females. Therefore, the discussion primarily reflects this population. Other restrictive eating disorders are beyond the scope of this review.

### Hypothalamic-pituitary-gonadal axis

Energy deprivation and reduced fat mass suppress the hypothalamic–pituitary–gonadal axis by impairing gonadotropin-releasing hormone (GnRH) pulsatility, leading to diminished luteinizing hormone (LH) and follicle-stimulating hormone (FSH) secretion and, consequently, reduced ovarian steroidogenesis. This results in hypoestrogenism and hypoandrogenism, disrupting normal reproductive function and causing anovulation, amenorrhea, or arrested puberty in affected females (2–4). The accompanying estradiol deficiency compromises bone accrual, lowers bone mineral density, and alters bone geometry during a period in which adolescents typically gain most of their peak bone mass. Androgen levels are likewise reduced, contributing further to impaired bone health (5). Relative androgen and estrogen deficiency has also been linked to greater severity of depressive and anxiety symptoms in women with AN (6, 7).

### Growth hormone-insulin-like growth factor-1 axis

Under physiological conditions, growth hormone (GH) stimulates hepatic insulin-like growth factor-1 (IGF-1) production, which mediates its anabolic actions on bone and muscle. In anorexia nervosa, GH levels are markedly elevated whereas IGF-1 levels are reduced, reflecting acquired GH resistance (8–10). Several mechanisms contribute to this GH–IGF-1 dysregulation. First, hypoinsulinemia associated with malnutrition decreases IGF-1 synthesis, as insulin is required for optimal GH signaling in the liver (11). Second, low leptin levels and alterations in other metabolic hormones (e.g., ghrelin, peptide YY) impair hypothalamic regulation of the GH–IGF-1 axis. Increased levels of ghrelin cause increased growth hormone secretion.

Third, increased cortisol secretion in AN may further suppress IGF-1 production (12). Clinically, low IGF-1 levels are strongly associated with reduced BMD, delayed puberty, and impaired longitudinal growth in adolescents with AN (8).

### Hypothalamic-pituitary-adrenal (HPA) axis

Cortisol secretion is regulated by the HPA-axis, in which corticotropin-releasing hormone (CRH) from the hypothalamus stimulates pituitary release of adrenocorticotropic hormone (ACTH), which in turn promotes adrenal cortisol synthesis. In AN, this regulatory system is typically hyperactive, producing sustained hypercortisolemia that promotes bone resorption, reduces muscle protein synthesis, and contributes to anxiety and depressive symptoms (2, 9). Excess cortisol also inhibits GnRH-secretion, thereby contributing to hypogonadotropic hypogonadism (9). Furthermore, as described earlier, elevated cortisol levels may suppress hepatic production of IGF-1 (12).

### Hypothalamic-pituitary-thyroid (HPT) axis

Patients with AN often present with biochemical and clinical features consistent with euthyroid sick syndrome. This condition is characterized by reduced peripheral conversion of thyroxine (T4) to its active form, triiodothyronine (T3), as an adaptive response to prolonged caloric restriction or chronic illness.

Suppression of the HPT-axis results in an attenuated TSH response despite low thyroid hormone levels, while malnutrition and reduced IGF-1 further contribute to decreased thyroid gland volume. Clinically, these endocrine alterations manifest as bradycardia, hypotension, and hypothermia, which are hallmarks of metabolic adaptation in undernourished states (2).

Thyroid hormones also play a critical role in skeletal metabolism by regulating osteoblast and osteoclast activity. Consequently, thyroid dysfunction in AN may further exacerbate bone mineral density loss (13). A recent study by Wronski et al. (14) reported a correlation between low T3 concentrations and the severity of depressive symptoms in individuals with AN. Although there is some interest in exploring low-dose thyroid hormone supplementation to address psychiatric comorbidities, current clinical guidelines advise against such intervention, as exogenous thyroid hormone replacement may interfere with this physiological adaptive response to energy deprivation (14).

## Clinical consequences of endocrine dysregulation

### Effects on growth

Linear growth is often compromised in children and adolescents with AN, particularly when the illness begins before or during the pubertal growth spurt. Chronic energy deficiency and GH resistance reduce IGF-1 bioactivity, leading to diminished height velocity and, in some cases, lower final adult stature. Although partial catch-up growth may occur after nutritional rehabilitation, meta-analytic evidence shows that recovery is frequently incomplete, especially in early-onset or long-standing illness (15). Rozé et al. identified prolonged hospitalization, a marker of illness severity, as the only significant predictor of reduced final height in early-onset AN (16). Modan-Moses et al. similarly reported that patients diagnosed before age 13 or shortly after menarche had more severely impaired height velocity, with incomplete height recovery despite weight restoration (17).

### Effects on puberty and menstrual function

AN represents approximately 0.9% of cases of pubertal delay cases in adolescents (3). Hypothalamic hypogonadism is a key endocrine feature and results in delayed puberty, primary amenorrhea, or secondary amenorrhea in up to 90% of affected girls. Even modest weight loss of 10%–15% can disrupt gonadotropin secretion and precipitate menstrual cessation (2). The resumption of menses is most strongly associated with weight restoration, increased body fat mass, and rising leptin levels. However, in some patients menstrual function remains impaired, suggesting persistent hypothalamic suppression or long-lasting alterations in GnRH pulsatility (18).

Several studies have examined predictors of these menstrual disturbances. Rozé et al. followed girls with early- or prepubertal-onset AN into adulthood and identified two independent predictors of delayed menarche: older age at illness onset (*p* = 0.0003) and lower minimum BMI during the course of illness (*p* = 0.004), indicating that both timing and nutritional severity shape pubertal development (16). Similarly, Dempfle et al. studied 172 adolescent girls with AN and found that 47% resumed menstruation within 12 months. Higher BMI at the beginning and end of treatment, greater weight gain, shorter amenorrhea duration, and later age at menarche were associated with menstrual recovery, which typically occurred once patients reached approximately 90%–95% of expected body weight (15th–20th BMI percentile) (19).

### Effects on bone health

The skeletal system is markedly compromised in AN due to the combined effects of hypoestrogenism, GH resistance with low IGF-1, hypercortisolemia, and altered appetite-regulating hormones, all of which shift bone turnover toward resorption (3, 4, 20). Patients with AN also show increased marrow adipose tissue (MAT), which is inversely related to BMD because it reflects a shift of mesenchymal stem cells toward adipogenic rather than osteoblastic differentiation (9). Clinically, osteopenia occurs in up to 90% of adult women with AN, while 30%–40% meet criteria for osteoporosis, with fracture risk increased up to sevenfold and particularly high in early-onset cases (20). Because adolescence is critical for achieving peak bone mass, skeletal deficits acquired during this period may be only partially reversible.

## Therapeutic approaches

### Growth

Recombinant human growth hormone (rhGH) has been explored as an adjunctive treatment for growth failure in adolescents with AN, yet its overall efficacy remains limited because hepatic GH resistance typically persists under malnourished conditions. In adults with AN, even supraphysiologic GH doses do not increase circulating IGF-1, although reductions in fat mass have been observed, likely due to GH-mediated lipolysis. GH and IGF-1 levels generally improve during refeeding, underscoring the primacy of nutritional rehabilitation for restoring growth-related endocrine function (2).

In contrast, pediatric studies have reported more favorable outcomes. The pilot study by Léger et al. (10) examined the impact of rhGH (daily subcutaneous injection of rhGH at a mean initial dose of 0.040 ± 0.006 mg/kg/day) on linear growth in adolescents with AN and severe growth retardation. The study included 10 adolescents with AN (mean age 13.5y). During treatment, a significant increase in growth velocity was observed, with mean annual height gain rising from +1.1 ± 0.8 cm/year before therapy to +4.2 ± 1.2 cm/year during rhGH administration (*p* < 0.01). These findings suggest that rhGH may stimulate linear growth in nutritionally compromised adolescents with AN, even in the context of persistent energy deficiency. Nevertheless, the study's small sample size and absence of a control group limit the generalizability of the results (10). Building on these findings, the same research group (21) conducted a randomized, placebo-controlled trial in younger children with anorexia nervosa and persistent growth failure (*N* = 14), demonstrating that GH therapy (0.050 mg/kg/day) significantly improved height velocity and IGF-1 concentrations after six months, although this effect diminished by twelve months and did not translate into gains in bone mineral density (21).

Overall, current evidence indicates that rhGH may transiently improve IGF-1 levels and certain growth parameters in malnourished children and adolescents with anorexia nervosa, but its effects are modest and temporary. Given the small and largely uncontrolled nature of existing studies, rhGH should be considered only as an adjunctive therapy. Nutritional rehabilitation remains the essential and most effective intervention, as restoration of adequate energy balance is the primary determinant of endocrine normalization and catch-up growth.

### Amenorrhea and pubertal delay

Endocrine recovery through hormone replacement therapy (HRT) has been explored through interventional studies.

A pilot study of Léger et al. explored the effects of rhGH on height velocity in 10 adolescent girls with AN, all of whom had severe growth failure and delayed puberty (10). They also observed pubertal progression in these patients. The participants received rhGH treatment for at least 18 months, with growth parameters and pubertal status monitored throughout treatment and follow-up. Over the two-year treatment period it was documented that 7 out of 10 girls achieved spontaneous menarche, with a median time of 2.6 years (range 1.3–2.9) from treatment initiation. However, the study does not include a control group, making it difficult to separate the effects of rhGH from natural recovery due to nutritional and weight stabilization.

A randomized placebo-controlled trial of Misra et al. (22) evaluated the effects of physiologic transdermal estrogen replacement in adolescent girls ages 12–18 years with AN (*N* = 110). Participants in the treatment arm received transdermal 17β-estradiol administered via patch at 0.025 mg/day, with the dose increased to 0.0375 mg/day after 6 months, along with oral micronized progesterone (200 mg for 10 days each month) to ensure endometrial protection. At baseline, no significant differences were observed between the estrogen-treated and placebo groups in terms of pubertal maturity, BMI or baseline bone mineral density parameters. Although the primary outcome was bone density, the study also examined menstrual recovery. After 18 months, 55% of adolescents receiving estrogen had resumed menses compared with 20% in the placebo group, suggesting that physiologic estrogen may help reactivate the HPG axis in partially weight-restored adolescents. Nevertheless, the authors emphasized that estrogen cannot replace nutritional rehabilitation, and that these findings must be interpreted in light of the inclusion of relatively weight-stable adolescents with persistent amenorrhea (22).

### Bone health

Estrogen therapy has been widely investigated in AN. Oral estrogen–progestin combinations consistently fail to improve BMD in adolescents, likely due to suppression of hepatic IGF-1 synthesis. However, Misra et al. (22) conducted a randomized, double-blind, placebo-controlled trial to assess the effect of physiologic estrogen replacement on BMD in adolescent girls with AN. Over an 18-month period, 110 girls with AN received transdermal 17β-estradiol (100 μg twice weekly) combined with cyclic progesterone if pubertally mature, or low-dose oral ethinyl estradiol (3.75 mg daily for the first 6 months, 7.5 mg daily for the second 6 months, and 11.25 mg daily for the last 6 months) if immature. Compared with placebo, estrogen-treated participants showed significant increases in lumbar spine and hip BMD Z-scores (*p* = 0.04), with gains comparable to those seen in healthy controls. Physiologic estrogen replacement did not suppress IGF-1 levels, preserving its anabolic influence on bone metabolism. These findings support the use of physiologic, rather than pharmacologic, estrogen replacement to promote bone health in adolescents with AN. However, the study was limited by a relatively short follow-up period and a high attrition rate, and long-term effects on final bone mass and reproductive recovery remain uncertain.

Building upon these findings, Singhal et al. conducted a double-blind, randomized, placebo-controlled trial to determine whether the addition of recombinant human insulin-like growth factor-1 (rhIGF-1) would provide additional skeletal benefits (5). Seventy-five adolescents and young women with anorexia nervosa received either transdermal estradiol alone or in combination with rhIGF-1 at a dose of 30–40 µg/kg administered subcutaneously twice daily for twelve months. Transdermal estradiol significantly increased lumbar spine bone mineral density compared with placebo (*p* = 0.004), but the addition of rhIGF-1 did not further enhance bone density.

Other anabolic strategies have yielded mixed results. DiVasta et al. found that combined dehydroepiandrosterone (DHEA) and oral estrogen-progestin therapy preserved femoral BMD and improved cortical geometry in young adults with AN (23). However, their later trial in adolescents showed no benefit and even declines in BMD among those with open growth plates, suggesting that response to DHEA–estrogen therapy is strongly dependent on skeletal maturity (24).

Bisphosphonates show some efficacy in adults with AN, but limited adolescent data and safety concerns preclude their routine use (25).

## Recommendations in treating adolescents with AN

### Diagnostic assessment

Initial evaluation should include a detailed personal and family medical history (including parental heights for calculation of mid-parental target height and parental pubertal history), pubertal staging, and a thorough somatic examination to identify the impact of malnutrition and to determine the need for inpatient management. Laboratory investigations should assess for metabolic and electrolyte abnormalities, endocrine changes, and other conditions mimicking AN (26).

### Nutritional rehabilitation and prevention of refeeding syndrome

Nutritional rehabilitation remains the cornerstone of treatment. Individualized weight targets are based on premorbid BMI, growth history, and pubertal stage, aiming to restore the child's pre-illness BMI trajectory (27). Approaches proposed in the literature include percentage of expected body weight (%EBW) based on CDC growth charts, with evidence suggesting that approximately two-thirds of adolescents resume menses at 95% EBW (4, 26).

Expected weight gain is 200–500 g/week in outpatients and 0.5–1.5 kg/week in inpatients. Initial caloric intake typically begins around 250 kcal/day above baseline, with gradual increases every few days. For children at high risk of refeeding syndrome, especially those with >20% weight loss or ECG abnormalities, the UK Medical Emergencies in Eating Disorders (MEED) guideline recommend cautious refeeding, starting at 5–10 kcal/kg/day under close monitoring (28).

Preventive phosphate supplementation (20 mg/kg/day, divided doses for at least one month) and correction of potassium and magnesium are essential to reduce refeeding syndrome risk. Vitamin D (100,000 IU every 3 months) and calcium (1,000–1,500 mg/day) are recommended to support bone mineralization (27).

### Pharmacological and hormonal treatment

To date, no pharmacological agent has proven effective for weight restoration or core symptom reduction in children or adolescents with AN.

Physiological transdermal 17β-estradiol replacement with cyclic oral progesterone (as described by Misra et al., 2011) may be considered to support bone health in partially weight-restored adolescents under endocrinological supervision (22).

### Psychological and family-based interventions

All major guidelines agree that family-based therapy (FBT) is the evidence-based first-line intervention for adolescents with AN. Cognitive-behavioral therapy (CBT-E) or multifamily therapy may be employed as adjuncts or second-line approaches. For younger children, parental involvement is particularly critical to re-establish normal eating patterns and reduce illness-related behaviors (27).

Both the NICE guidelines (29) and AAP guidelines (26) stress that diagnosis and management of AN in children should occur within a multidisciplinary specialist team with expertise in pediatric and mental health care. They both emphasize early intervention, nutritional restoration, and psychological treatment as first-line care, recommending FBT as the treatment of choice for children and adolescents.

### Follow-up and long-term care

Ongoing monitoring should continue for at least 12 months after remission. Clinical review should include weight, height, vital signs, psychological status, and laboratory parameters. Bone age assessment is warranted in cases of delayed growth or puberty, while bone densitometry is indicated every two years or as clinically necessary. Educational reintegration should be encouraged, and care continuity ensured through coordination between pediatric, psychiatric, and educational professionals.

## Conclusion

Endocrine dysfunction in adolescent anorexia nervosa represents a complex adaptive response to chronic energy deprivation, manifesting as hypogonadotropic hypogonadism, growth hormone resistance, hypercortisolemia, and thyroid suppression. These alterations disrupt pubertal progression, impair growth, and compromise skeletal development during a critical period of maturation.

Nutritional rehabilitation remains the primary determinant of endocrine and reproductive recovery. Evidence supports physiologic transdermal estrogen replacement as the most effective adjunctive therapy for improving bone mineral density and promoting menstrual resumption in partially weight-restored adolescents. Conversely, oral estrogen or combined oral contraceptives offer limited or inconsistent benefit. Recombinant growth hormone and IGF-1 administration may transiently improve growth or bone markers but fail to produce sustained skeletal gains, highlighting the dominance of nutritional and metabolic restoration over pharmacologic manipulation.

Future research should focus on individualized, age-appropriate interventions, incorporating hormonal replacement only after nutritional stabilization and under strict endocrinological supervision. Long-term studies are needed to assess the safety, efficacy, and reproductive outcomes of emerging therapies such as leptin analogues and bone-active agents. Ultimately, a multidisciplinary approach integrating nutritional, psychological, and endocrine management remains essential to optimize somatic and reproductive outcomes in adolescents with anorexia nervosa.

## References

[1] Keski-Rahkonen A Raevuori A Hoek HW. Epidemiology of Eating Disorders: An Update. Oxford: Oxford University Press (2018).

[2] Usdan LS Khaodhiar L Apovian CM. The endocrinopathies of anorexia nervosa. Endocr Pract. (2008) 14(8):1055–63. 10.4158/EP.14.8.1055 19095609 PMC3278909

[3] Simão Raimundo D Figueiredo C Raposo A Dias Pereira B. Arrested puberty in an adolescent male with anorexia nervosa successfully resumed with multidisciplinary care. Case Rep Pediatr. (2021) 2021:5512532. 10.1155/2021/5512532 34336338 PMC8294969

[4] Golden NH Katzman DK Sawyer SM Ornstein RM Rome ES Garber AK Update on the medical management of eating disorders in adolescents. J Adolesc Health. (2015) 56(4):370–5. 10.1016/j.jadohealth.2014.11.020 25659201

[5] Singhal V Bose A Slattery M Haines MS Goldstein MA Gupta N Effect of transdermal estradiol and insulin-like growth factor-1 on bone endpoints of young women with anorexia Nervosa. J Clin Endocrinol Metab. (2021) 106(7):2021–35. 10.1210/clinem/dgab145 33693703 PMC8427708

[6] Kimball A Schorr M Meenaghan E Bachmann KN Eddy KT Misra M A randomized placebo-controlled trial of low-dose testosterone therapy in women with anorexia Nervosa. J Clin Endocrinol Metab. (2019) 104(10):4347–55. 10.1210/jc.2019-00828 31219558 PMC6736210

[7] Schorr M Miller KK. The endocrine manifestations of anorexia nervosa: mechanisms and management. Nat Rev Endocrinol. (2017) 13(3):174–86. 10.1038/nrendo.2016.175 27811940 PMC5998335

[8] Misra M Aggarwal A Miller KK Almazan C Worley M Soyka LA Effects of anorexia nervosa on clinical, hematologic, biochemical, and bone density parameters in community-dwelling adolescent girls. Pediatrics. (2004) 114(6):1574–83. 10.1542/peds.2004-0540 15574617

[9] Singhal V Misra M Klibanski A. Endocrinology of anorexia nervosa in young people: recent insights. Curr Opin Endocrinol Diabetes Obes. (2014) 21(1):64–70. 10.1097/MED.0000000000000026 24275621 PMC3971868

[10] Léger J Fjellestad-Paulsen A Bargiacchi A Doyen C Ecosse E Carel JC Can growth hormone treatment improve growth in children with severe growth failure due to anorexia nervosa? A preliminary pilot study. Endocr Connect. (2017) 6(8):839–46. 10.1530/EC-17-0200 29038330 PMC5682412

[11] Misra M Klibanski A. Endocrine consequences of anorexia nervosa. Lancet Diabetes Endocrinol. (2014) 2(7):581–92. 10.1016/S2213-8587(13)70180-3 24731664 PMC4133106

[12] Jada K Djossi SK Khedr A Neupane B Proskuriakova E Mostafa JA. The pathophysiology of anorexia nervosa in hypothalamic endocrine function and bone metabolism. Cureus. (2021) 13(12):e20548. 10.7759/cureus.20548 35103128 PMC8776521

[13] Donaldson AA Gordon CM. Skeletal complications of eating disorders. Metab Clin Exp. (2015) 64(9):943–51. 10.1016/j.metabol.2015.06.007 26166318 PMC4546560

[14] Wronski ML Tam FI Seidel M Mirtschink P Poitz DM Bahnsen K Associations between pituitary-thyroid hormones and depressive symptoms in individuals with anorexia nervosa before and after weight-recovery. Psychoneuroendocrinology. (2022) 137:105630. 10.1016/j.psyneuen.2021.105630 34959165

[15] Neale J Pais SMA Nicholls D Chapman S Hudson LD. What are the effects of restrictive eating disorders on growth and puberty and are effects permanent? A systematic review and meta-analysis. J Adolesc Health. (2020) 66(2):144–56. 10.1016/j.jadohealth.2019.08.032 31771922

[16] Rozé C Doyen C Le Heuzey MF Armoogum P Mouren MC Léger J. Predictors of late menarche and adult height in children with anorexia nervosa. Clin Endocrinol (Oxf). (2007) 67(3):462–7. 10.1111/j.1365-2265.2007.02912.x 17561975

[17] Modan-Moses D Yaroslavsky A Kochavi B Toledano A Segev S Balawi F Linear growth and final height characteristics in adolescent females with anorexia nervosa. PLoS One. (2012) 7(9):e45504. 10.1371/journal.pone.0045504 23029058 PMC3445517

[18] Fontana L Garzia E Marfia G Galiano V Miozzo M. Epigenetics of functional hypothalamic amenorrhea. Front Endocrinol (Lausanne). (2022) 13:953431. 10.3389/fendo.2022.953431 36034425 PMC9415998

[19] Dempfle A Herpertz-Dahlmann B Timmesfeld N Schwarte R Egberts KM Pfeiffer E Predictors of the resumption of menses in adolescent anorexia nervosa. BMC Psychiatry. (2013) 13:308. 10.1186/1471-244X-13-308 24238469 PMC3832684

[20] Resulaj M Polineni S Meenaghan E Eddy K Lee H Fazeli PK. Transdermal estrogen in women with anorexia nervosa: an exploratory pilot study. JBMR Plus. (2020) 4(1):e10251. 10.1002/jbm4.10251 31956852 PMC6957987

[21] Léger J Fjellestad-Paulsen A Bargiacchi A Pages J Chevenne D Alison M One year of GH treatment for growth failure in children with anorexia Nervosa: a randomized placebo-controlled trial. J Clin Endocrinol Metab. (2021) 106(7):e2535–46. 10.1210/clinem/dgab203 33772303

[22] Misra M Katzman D Miller KK Mendes N Snelgrove D Russell M Physiologic estrogen replacement increases bone density in adolescent girls with anorexia nervosa. J Bone Miner Res. (2011) 26(10):2430–8. 10.1002/jbmr.447 21698665 PMC3304439

[23] DiVasta AD Feldman HA Beck TJ LeBoff MS Gordon CM. Does hormone replacement normalize bone geometry in adolescents with anorexia nervosa? J Bone Miner Res. (2014) 29(1):151–7. 10.1002/jbmr.2005 23744513 PMC3812374

[24] DiVasta AD Feldman HA O'Donnell JM Long J Leonard MB Gordon CM. Impact of adrenal hormone supplementation on bone geometry in growing teens with anorexia nervosa. J Adolesc Health. (2019) 65(4):462–8. 10.1016/j.jadohealth.2019.04.003 31227390 PMC7001735

[25] Peebles R. Adolescents are not just smaller adults: consideration of growth plates and pubertal stage when addressing bone mineral density in adolescents with anorexia nervosa. J Adolesc Health. (2019) 65(4):435–7. 10.1016/j.jadohealth.2019.07.019 31542108

[26] Hornberger LL Lane MA, COMMITTEE ON ADOLESCENCE. Identification and management of eating disorders in children and adolescents. Pediatrics. (2021) 147(1):e2020040279. 10.1542/peds.2020-040279 33386343

[27] Ayrolles A Clarke J Godart N André-Carletti C Barbe C Bargiacchi A Early-onset anorexia nervosa: a scoping review and management guidelines. J Eat Disord. (2024) 12(1):182. 10.1186/s40337-024-01130-9 39558193 PMC11572092

[28] Royal College of Psychiatrists. Medical Emergencies in Eating Disorders: Guidance on Recognition and Management. London: Royal College of Psychiatrists (2022).

[29] National Institute for Health and Care Excellence (NICE). Eating disorders: recognition and treatment (NG69). Published online 2020. 33689256

